# Selective vulnerability of ARID1A deficient colon cancer cells to combined radiation and ATR-inhibitor therapy

**DOI:** 10.3389/fonc.2022.999626

**Published:** 2022-09-30

**Authors:** Shan Xu, Ali Sak, Ben Niedermaier, Yasin Bahadir Erol, Michael Groneberg, Emil Mladenov, MingWei Kang, George Iliakis, Martin Stuschke

**Affiliations:** ^1^ Strahlenklinik, Universitätsklinikum Essen, Essen, Germany; ^2^ Department of General Surgery, Mianyang Fulin Hospital, Mianyang, China

**Keywords:** colorectal cancer, SWI/SNF, synthetic lethality, ARID1A, ATR

## Abstract

ARID1A is frequently mutated in colorectal cancer (CRC) cells. Loss of ARID1A function compromises DNA damage repair and increases the reliance of tumor cells on ATR-dependent DNA repair pathways. Here, we investigated the effect of ionizing radiation (IR), in combination with ATR inhibitors (ATRi) in CRC cell lines with proficient and deficient ARID1A. The concept of selective vulnerability of ARID1A deficient CRC cells to ATRi was further tested in an *ex vivo* system by using the ATP-tumor chemosensitivity assay (ATP-TCA) in cells from untreated CRC patients, with and without ARID1A expression. We found selective sensitization upon ATRi treatment as well as after combined treatment with IR (P<0.001), especially in ARID1A deficient CRC cells (P <0.01). Knock-down of ARID1B further increased the selective radiosensitivity effect of ATRi in ARID1A negative cells (P<0.01). Mechanistically, ATRi abrogates the G2 checkpoint (P<0.01) and homologous recombination repair (P<0.01) in ARID1A deficient cells. Most importantly, *ex-vivo* experiments showed that ATRi had the highest radiosensitizing effect in ARID1A negative cells from CRC patients. Collectively, our results generate pre-clinical and clinical mechanistic rationale for assessing ARID1A defects as a biomarker for ATR inhibitor response as a single agent, or in a synthetic lethal approach in combination with IR.

## Introduction

Colorectal cancer (CRC), one of the most frequently occurring cancers, is still one of the main causes of cancer-associated mortality globally (1). Typically, CRC results from successively acquired genetic and epigenetic alterations, mainly related to proto-oncogene activation and loss of tumor suppressor function (2, 3). Standard treatments have failed so far to significantly improve the survival of CRC patients, and as a result novel, targeted and more selective therapy options are urgently needed. To this end, it is important to characterize genes associated with CRC genesis and progression in order to improve molecular diagnosis and the customized treatment of CRC (4).

ARID1A (also known as BAF250A) belongs to the SWI/SNF complex subunits (5). The SWI/SNF is an ATPase chromatin remodeling/tumor suppressor complex that modulates lineage-specific transcription through nucleosome remodeling, and also supports DNA repair by recruiting proteins to the damage sites (6). As a result, loss-of-function of SWI/SNF complex accounts for the genomic instability frequently associated with tumor development (7). Preclinical studies suggest that mutations in components of the SWI/SNF complex alter tumor biology, and may enhance its radio/chemotherapy and anticancer immune responses (8).

Among SWI/SNF complex subunits, ARID1A shows the greatest mutation frequency (10–40% loss-of-function) in human malignancies (9–11). ARID1A^-^ correlates with tumor aggressiveness and is a negative prognostic marker of treatment outcome (12). The mutation frequency of ARID1A within CRC is particularly high, especially in the subset of tumors displaying microsatellite instability (9). Considering that the mutation frequency of ARID1A is high in CRC, it is important to develop drugs for the selective targeting of ARID1A^-^ cancer cells and integrate them with standard therapies to develop novel treatment options. ARID1B, a paralog of ARID1A (13), has been recently recognized as a putative lethal target for ARID1A-mutant cancers, as ARID1B depletion impairs growth and destabilizes the SWI/SNF complex, which subsequently increases cell radiosensitivity (14, 15). In addition, ARID1A knock-down impairs repair of DSBs and generates dependence on ATR- or PARP- dependent damage processing in tumors cells, offering thus options for synthetic lethal interactions (16, 17).

Several studies have shown that ARID1A dependent synthetic lethality can be attained through diverse molecular mechanisms (18). Especially, ATR inhibition was suggested to be a synthetic lethal partner of ARID1A deficiency (17). To evaluate the synthetic lethality concept of ATR inhibition and ARID1A deficiency in colon cancer cell lines, the ATR inhibitor VE821 and VE822 were used to exert synergistic effects with irradiation on ARID1A^-^ cancer cells. We also tested the effect of ATR inhibitors in combination with ARID1B silencing for radiosensitization in CRC cells, with and without ARID1A deficiency (14).

## Materials and methods

### Cell lines

The human CRC cell lines with mutant (LS180, RKO, SW48) and wild-type (HCT15, HCT116 and Colo320DM) ARID1A were obtained from ATCC (LGC Standards, Wesel, Germany) and were designated as ARID1A^-^ and ARID1A^+^ cells, respectively. LS180, RKO and SW48 cells were grown in MEM (Invitrogen, ThermoFisher Scientific, Waltham, Massachusetts, USA) supplemented with 15% fetal bovine serum (FBS), 1% essential amino acids and antibiotics. HCT15, HCT116 and Colo320DM cells were grown in RPMI (Invitrogen) supplemented with 10% FBS and 1% antibiotics. U2OS and A549 cells harboring reporters for HR (DR-GFP) were grown as a monolayer in McCoy’s 5A medium supplemented with 10% FBS and antibiotics. All cells were maintained at 37°C in 5% CO_2_. Irradiation was carried out using a RS320 X-Ray machine (XStrahl Ltd, Walsall, UK) operating at 300 kV, 10 mA, at a dose rate of 0,9 Gy/min.

### Knock-down with siRNA

Cells were seeded in 6-well plates and incubated for about 20 hours, aiming at a 70–80% confluent cell monolayer. Cells were then washed in Hanks Balanced Salt Solution (HBSS) and OptiMEM (both Gibco), and subsequently incubated with transfection reagent for 4 h. We used 500 μl OptiMEM with 40 nM siRNA and 6 μl Lipofectamine RNAiMAX (Invitrogen, ThermoFisher Scientific) as a transfection reagent. To downregulate ARID1A, the siRNA s15786 was used at 40 nM. As controls, non-targeting siRNA (4390843 Ambion, ThermoFisher Scientific), as well as lipofectamine alone were used. After 4 h of incubation with the transfection reagent, 500 µl of culture medium containing twice the normal FBS concentration was added and cells were incubated for 48 h until harvesting for analysis. The expression of the targeted proteins was regularly checked by western blot.

### Immunoblotting

Western blots were performed with anti-ARID1A (Cell Signaling Technology, 12354P, Danvers, Massachusetts, USA) and anti-GAPDH (ab8245, Abcam, Cambridge, UK) antibodies. The secondary antibodies were HRP-linked raised against mouse or rabbit IgG (NA931V and NA934V, GE Healthcare, Chicago, Illinois, USA), and Alexa Fluor 488-linked antibodies raised against mouse IgG (A11029, Invitrogen, ThermoFisher Scientific).

### Clonogenic survival assay

48 h after transfection, cells were harvested and plated in triplicates in 9,6 cm^2^ 6-well culture dishes. After 4–6 h in culture, cells were irradiated and subsequently incubated for 10–14 days at 37°C in 5% CO_2_. Cells were fixed and stained with 96% ethanol, 15% Giemsa and destained with distilled water. Colonies consisting of at least 50 cells were counted. Surviving fractions after the indicated treatments are presented as the ratio of colonies scored in irradiated versus non-irradiated cells.

SF = number of colonies after IR/number of colonies in non-irradiated cells

### Immunofluorescence (IF) assay

For IF processing, cells were cultured directly on coverslips. To specifically detect IR-induced repair foci in G2-phase, cells in the exponential growth phase were subject to 30-min pulse-labeling using EdU (10 µM) immediately prior to irradiation. Subsequently, cells were subjected to 15-min 2% paraformaldehyde (PFA) fixation at specific time points followed by PBS (0.01 M phosphate buffer, 0.14 M NaCl, pH 7.0) washing and permeabilization (5-min in 0.5% Triton X-100, 50 mM EDTA and 100 mM Tris–HCl). After another PBS washing, cells were blocked with PBG buffer (consisting of 0.5% BSA fraction V and 0.2% Gelatin dissolved in PBS) at 4°C overnight. To detect the RAD51 protein, a mouse anti-RAD51 monoclonal antibody (mab, clone 14B4, GeneTex) was used. To detect γ-H2AX, a mouse anti-γ-H2AX mab (clone 3F2, Abcam) was used. Cells were subjected to 1.5 h incubation with primary antibodies, followed by PBS rinsing for 5 min thrice. Subsequently, a secondary anti-rabbit antibody conjugated to Alexa Fluor 568-labeled IgG or anti-mouse Alexa Fluor 488-labeled IgG (ThermoFisher) was added for 1 h. Thereafter, slides were developed using the Click-IT staining kit (ThermoFisher Scientific) in line with EdU detection protocols. Finally, coverslips were rinsed with PBS and incubated for 15 min in DAPI solution (0.1 µg/ml) before embedding, using Prolong Gold Antifade mounting medium, in microscope slides (ThermoFisher Scientific). A Leica TCS SP5 confocal microscope was used for detecting repair foci. Spillover from other channels was eliminated using sequential scanning. To allow comparison between different experiments, detector settings and antibody batch were kept constant.

### Analysis of digital images

To analyze 3D image stacks obtained after scanning in the confocal microscope, the Spots and Split Spots module in Imaris 8.0 software (Bitplane), or the Cell module in Imaris 8.0-9.3 software for determining the number of foci, were utilized. A constant threshold value for grayscale was used to separate background from signal within diverse experiments using the same antibody batches. Objects above a diameter of 0.5 µm following thresholding were scored as foci. About 150 cells at each time point and dose were scored. To restrict analysis to cells irradiated in G2-phase, foci were scored only in EdU negative cells. Data acquired by the Imaris software were further compiled and organized using the Orange graphic software.

### Quantitative image-based cytometry (QIBC)

RAD51 and γH2AX foci were also analyzed by IF in the different phases of the cell cycle using a high-throughput slide-scanner (AxioScan.Z1, Zeiss). EdU was used again to label S-phase cells as described above. Following irradiation, cells were fixed at specific time points, placed on coverslips and scanned in areas of 4 × 4 mm. In this way, 10.000–20.000 cells were scanned and were subsequently analyzed using Imaris to obtain estimates of RAD51 or γH2AX foci number in different phases of the cell cycle - determined by parallel analysis of EdU and DAPI staining. Data produced by Imaris were compiled for presentation using the format used during flow cytometric analysis (Kaluza, Beckman Coulter). Only cells in G2/M-phase during irradiation and post-irradiation incubation were analyzed.

### Analysis of HR using GFP reporter cell lines and I-SceI-induced DSBs

In these experiments, 2 × 10^6^ DR-GFP- A549 or DR-GFP- U2OS cells were subjected to ARID1A knock-down and 24 h later were transfected by nucleofection (Lonza) with 2 µg of I-SceI expressing plasmid pCMV3xNLS-I-SceI. After 24 h of additional incubation, cells were harvested by trypsinization and analyzed by flow cytometry (Gallios, Beckman Coulter) for GFP expression using a flow cytometer equipped with a 488-nm argon laser. GFP emission was collected using a 510BP filter at FL1. Repair efficiency was determined as the frequency of GFP-positive cells. Replicate cultures transfected with a GFP-expressing construct (pEGFP-N1, 1 µg/µ1 × 10^6^ cells) were used to determine transfection efficiency in each experiment. Only experiments with a transfection efficiency above 80% were analyzed.

### Ethical statement

All patients provided written informed consent that their information will be stored and used in the MianYang Fulin hospital database. Study approval was obtained from the independent ethics committee of the MianYang Fulin hospital, China (IRB ID: TJ-C20210701). The study was undertaken in accordance with the ethical standards of the World Medical Association Declaration of Helsinki. All excised samples were obtained from tumor tissues within 1 h after surgery. For each specimen, half of the material was sent to the laboratory for ATP-TCA Test, and the remainder was fixed with formalin for immunohistochemistry (IHC).

### Patient information and tissue specimens

This study was conducted using a total of 41 archived paraffin-embedded primary CRC samples. All patients underwent resection of primary tumors between July 2021 and January 2022 at MianYang Fulin hospital, China. None of the patients had preoperative chemotherapy or preoperative radiotherapy. The staging of tumors was determined according to the American Joint Committee on Cancer (AJCC) TNM staging system. Each tumor was pathologically classified according to the World Health Organization classification criteria.

### IHC and scoring

We analyzed ARID1A expression in 41 primary CRC tissues by means of IHC as previously reported (19). Briefly, we processed each tissue section by deparaffinage, rehydration, blocking of endogenous peroxide and antigen retrieval, followed by overnight incubation at 4°C with ARID1A (PSG3) antibody (sc-32761, Santa Cruz Biotechnology, Inc., CA, United States). Sections were rinsed with PBS containing 0.1% Tween-20, followed by 30-min incubation with anti-mouse secondary antibody, and were subsequently incubated with streptavidin-HRP. Diaminobenzidine tetrahydrochloride was employed for color development followed by hematoxylin counterstaining. All immunostained sections were examined and scored by 2 reviewers, blinded to the pathological and clinical data of the patients. We examined 1000 or more cancer cells on every slide and measured the level of staining and the percentage of stained cells. We classified immunostained sections as positive (>60% positive cells) and negative (≤60% positive cells) for ARID1A expression.

### ATP-TCA

ATP-TCA was conducted as previously described (20). We minced solid tumor tissues and treated with collagenase overnight (1.5 mg/ml, Sigma, Poole, UK; C8051). Later, Ficoll-Hypaque (Sigma; 1077-1) was used to remove excessive debris and red blood cells and cells were resuspended in complete medium without serum (CAM; DCS Innovative Diagnostik Systeme, Hamburg, Germany) containing gentamicin (Sigma), penicillin–streptomycin (Sigma), metronidazole (Rhône Poulenc Rorer, Eastbourne, UK) and amphotericin B (Sigma). Cell number and cell viability were determined by trypan blue exclusion and a cell suspension was prepared at ~200.000 cells/ml for solid tumors, or ~100.000 cells/ml for malignant effusions. Polypropylene, round-bottomed 96-well plates (Corning-Costar, High Wycombe, UK) were prepared with CAM and inhibitors using six dilutions (6.25–200%) in triplicate. The drug concentration (TDC) range was previously determined from pharmacokinetic and biological response data. All drug solutions were prepared and stored according to the manufacturer’s instructions. Dilutions were prepared from freshly made working solutions up to 800% the TDC. Combinations of drugs were tested by adding both drugs at their 800% TDC. Two reference rows were also prepared in each plate, including medium only (MO) of CAM without drug, and maximum inhibitor (MI) concentration for full killing and zero ATP count were determined. After 6 days of incubation at 37°C, 5% CO2 and 100% humidity, the detergent-based Tumor Cell Extraction Reagent (DCS Innovative Diagnostik Systeme) was used to lyse cells and determine ATP levels in a microplate luminometer using the luciferin–luciferase assay (MPLX; Berthold, Pforzheim, Germany).

### Statistical analysis

All statistical analyses were performed using SPSS 14.0 or SAS (version 14.1, SAS Institute, Cary, NC, US) statistical software. A p value < 0.05 was considered statistically significant. The correlation between ARID1A expression and clinicopathological characteristics was analyzed by χ^2^ test, or Kruskal-Wallis H test, depending on the data analyzed. The correlation between radiation dose response and/or ATRi in ARID1A+ versus ARID1A- cells was determined as modification of the linear term of the linear-quadratic model. ANOVA was used to calculate the statistical significance between two groups at a given radiation dose. IC50 values and graphics were done using GraphPad Prism 9 software using the inhibitor vs normalized response method.

## Results

### ATR inhibitors selectively radiosensitize CRC cells with mutant ARID1A

**Figure 1:**
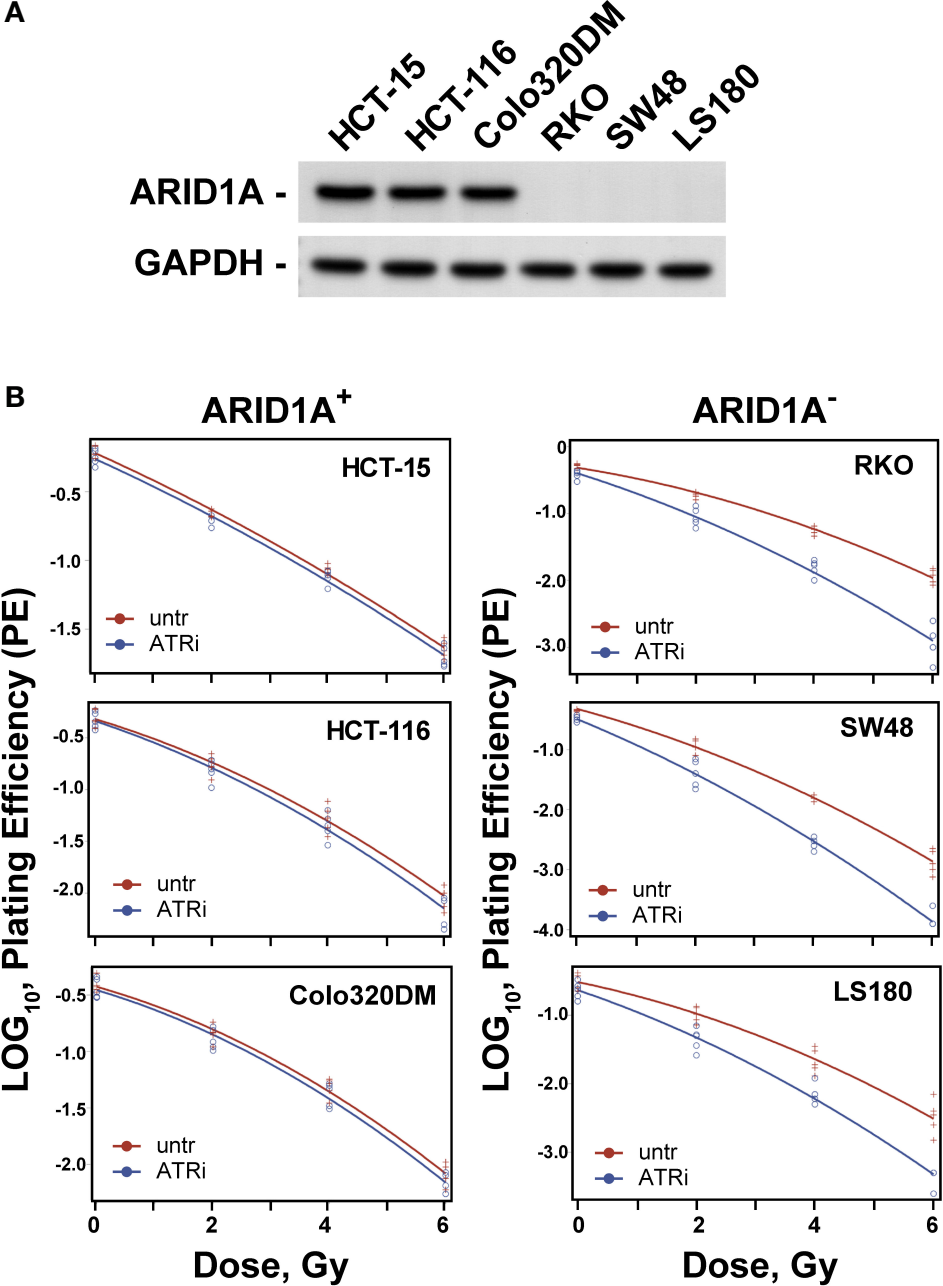
Effect of ATRi on radiosensitivity. **(A)** ARID1A expression in colon cancer lines; **(B)** Effect of ATRi (VE822) on radiosensitivity. ARID1A+ and ARID1A- cell lines were pre-treated for 1 h with 20 nM VE822 and irradiated with 0 Gy, 2 Gy, 4 Gy and 6 Gy. Plating efficiency of sham treated (untr) and VE822 treated (ATRi) cells were plotted as log10 for ARID1A^+^ and ARID1A^-^ cell lines. Results of 3 independent experiments are shown for CRC cell lines.

Colorectal carcinoma cell lines with wild-type ARID1A (HCT15, HCT116, Colo320DM) and mutant ARID1A (RKO, SW48, LS180) were used. The expression of ARID1A was examined in all cell lines by immunoblotting (**Figure 1A**). The data clearly show that cells with mutant ARID1A have no expression of ARID1A and thus designated ARID1A deficient (ARID1A^-^) and proficient (ARID1A^+^). The effect of ATR inhibitors (VE821 and VE822) in combination with ionizing radiation was tested on the clonogenic survival of these CRC cell lines. As a first step, the concentration of VE821 and VE822 for 50% survival (IC50) was determined for each cell line by using the colony formation assay and the results are summarized in **Supplemental Table S1**. The IC50 values for VE821 and VE822 of 20.0 ± 1.0 nM and 1.3 ± 0.2 µM was significantly (P<0.001) lower for CRC cell lines with mutant ARID1A as compared to 88.4 ± 11.4 nM and 4.8 ± 1.8 µM for cell lines with wild-type ARID1A.

For evaluation of the inhibitor-mediated radiosensitization, the surviving fraction after exposure to 2 Gy (SF2) alone was first determined for all cell lines. As shown in **Supplemental Table S2**, there is a trend for lower SF2 values in ARID1A^-^ cells (P>0.05). Next, cells were treated with ATR inhibitors VE821 and VE822 deficient cell lines at the above determined IC50 concentrations i.e., 1 µM and 20 nM for 1 h, before exposure to 0 Gy or 2 Gy and the surviving fraction was determined. Both, VE821 (P<0,01) and VE822 (P<0,001) radiosensitized ARID1A^-^ cells more efficiently than ARID1A^+^ cells (**Supplemental Table S2**). Because of its higher effectiveness, the ATR inhibitor VE822 was used for further experiments with additional irradiation doses of 4 Gy and 6 Gy to obtain full dose-effect relations for all cell lines. ARID1A^+^ and ARID1A^-^ cell lines were pre-treated for 1 h with 20 nM VE822 and irradiated with 0 Gy, 2 Gy, 4 Gy and 6 Gy. The radiosensitizing effect of VE822, as quantitated by the dose modifying factor (DMF), was highly significant (P<0,0001) for ARID1A^-^ cells but not for ARID1A competent cell lines (**Figure 1B**; **Supplement Figure S1** and **Table 1**). In addition, ARID1A^+^ cell lines were also exposed to a concentration of 90 nM V822, but the slope of the linear term of the survival curves did not change (p>0.4 for all three ARID1A^+^ cell lines) (**Supplement Figure S2**).

**Table 1:** Radiosensitizing effect of ATRi (VE822) for ARID1A^+^ and ARID1A^-^ colon cancer cell lines from the clonogenic assay.

Cell line	Dose modifying factor (DMF)	P
HCT15 (ARID1A^+^)	0.995 (95% CI:0.959-1.032)	0.78
HCT116 (ARID1A^+^)	0.962 (95% CI:0.894-1.036)	0.30
Colo320DM (ARID1A^+^)	0.981(95% CI:0.924-1.041)	0.51
SW48 (ARID1A^-^)	0.726 (95% CI:0.669-0.790)	<0.0001
RKO(ARID1A^-^)	0.721 (95% CI:0.664-0.783)	<0.0001
LS180 (ARID1A^-^)	0.766 (95% CI:0.691-0.849)	<0.0001

Colony data were analyzed using a linear-quadratic model describing the dependence of the logarithm of cellular survival on dose. The interaction between ATRi and the radiation dose response was described as a slope modifying effect of the linear term of the linear-quadratic model. ANOVA was used to calculate the statistical significance between groups.

Since the cell lines were not isogenic and thus have different gene expression profiles, it is unclear how these heterogeneities among different cell lines influence the observed results and if this only depends on the ARID1A status. Therefore, the effect of ATRi after the knock-down of ARID1A expression was evaluated in two different ARID1A^+^ CRC cell lines (HCT15 and HCT116) to assess the effect of different genetic background on the treatment response (**Supplement Figure S3**). The treatment of ARID1A^+^ cell lines with 20 nM V822 and control siRNA had no effect on the viability after irradiation. However, ARID1A knock-down led to a significant (P<0.01) reduction of the viability in both ARID1A^+^ CRC cells. In addition, knock-down of ARID1A combined with VE822 enhanced the radiosensitivity of ARID1A^+^ CRC cells (P<0.001). These results were consistent with our previous observations that ARID1A^-^ CRC cell lines are more sensitive to ATR inhibitors and indicated that the radiosensitizing effect after ATRi treatment mainly relied on the ARID1A status of the cell lines.

In order to test the possibility that ATRi would further potentiate the radiation sensitivity of CRC cells after knock-down of ARID1B, a synthetic lethal partner of ARID1A, cells were treated with VE822 after transfection with siRNA targeting ARID1B. For these experiments two ARID1A^-^ (RKO, SW48) and two ARID1A^+^ (HCT15 and HCT116) cell lines were used. Cell lines were first transfected with siRNA for 48 h and treated subsequently with the respective IC50 concentrations (1 µM for VE821 and 20 nM for VE822) for ARID1A^-^ cells; cells were irradiated 1 h later with 2 Gy and the respective surviving fraction determined. The results demonstrate higher radiosensitization after knock-down of ARID1B in ARID1A^-^ CRC cells (**Supplement Figure S4**; **Supplement Table S2**) and confirm our previous results of the synthetic lethality concept of ARID1A and ARID1B (14). Interestingly, the radiosensitizing effect on SF2 of both ATR inhibitors was even higher compared to the ARID1A effect (**Supplemental Table S2**). Overall, the presented data showed that ATRi significantly (P<0.05) potentiates the synthetic lethality effect of ARID1B knock-down in ARID1A deficient CRC cell lines (**Supplemental Figure S5**; **Supplemental Table S2**).

### Cell cycle specific radiosensitization of CRC cell lines by ATRi

**Figure 2:**
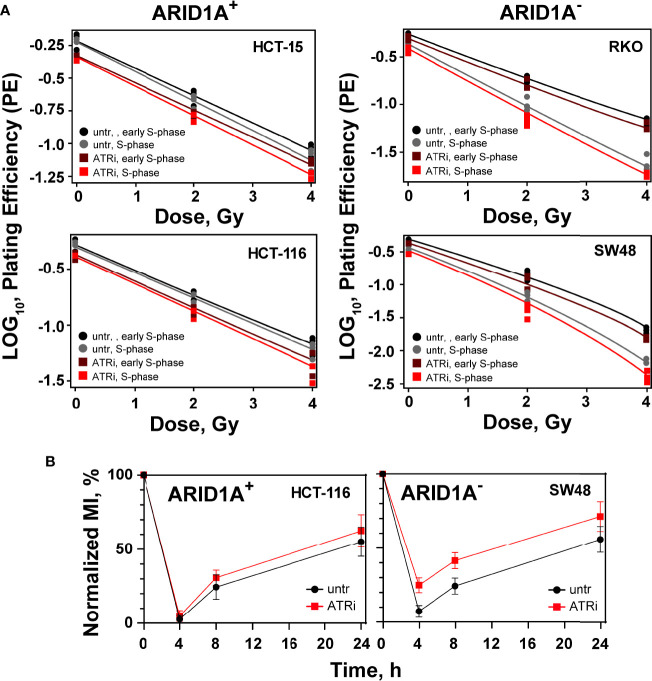
Cell cycle effect of ATRi/ARID1A. **(A)** Synchronized cells in early S phase and mid S phase were pre-treated for 1 h with VE822 and irradiated thereafter with 0 Gy, 2 Gy and 4 Gy. Plating efficiency of sham treated (untr) and VE822 treated (ATRi) cells were plotted as log10 for ARID1A^+^ and ARID1A^-^ cell lines. **(B)** Exit from G2 phase into the M phase was measured after treatment with VE822 and irradiation in ARID1A^-^ SW48 and ARID1A^+^ HCT116 cell lines. Fraction of phospho-histone H3 positive cells were plotted against time after irradiation. MI = phospho-histone H3 in radiation/phospho-histone H3 in no-radiation × 100%. Results of 3 independent experiments are shown for CRC cell lines.

ATR is a key component of the intra S-phase and G2 -phase cell cycle checkpoints that are activated by resection at DSBs, mainly taking place in S- and G2-phases of the cell cycle. We investigated therefore the cell cycle specific effect of ATRi on the radiosensitization of ARID1A^-^ CRC cell lines. Treatment with aphidicolin for 20 h synchronize cells at late G1/early S-phase stage of the cell cycle; cells are at mid S-phase 6 h after release from the aphidicolin block. The cell cycle distribution at 0 h and 6 h after release from the aphidicolin block is shown in **Supplemental Figure S6**. It is evident that 80-90% of cells were in the S- and G2-phases of the cell cycle at 6 h after release from aphidicolin block. We tested the effect of ATRi in early S phase (0 h) versus mid S-phase (6 h) in two ARID1A^-^ cell lines (RKO and SW48), as well as in two ARID1A+ cell line (HC15 and HCT116) (**Figure 2**; **Supplemental Figure S7** and **Table S3**). Treatment with ATRi significantly (P<0.0001) reduced the surviving fraction of ARID1A^-^ but not of ARID1A^+^ cell lines when irradiated at early as well as in mid S-phase (**Figure 2**; **Table S3**). The respective dose modifying factors for radiosensitization by ATRi are depicted in **Supplemental Table S3**. The present data show a tendency towards higher DMF in mid S-phase compared to early S-phase cells in both cell lines.

### ATRi abrogates IR induced G2/M checkpoint in ARID1A^-^ CRC cells

ATR is a principal mediator of the G2/M cell cycle checkpoint, which prevents the premature entry of cells with DNA damage into mitosis (18). Thus, inhibition of ATR will abrogate the G2/M cell cycle checkpoint and will increase the sensitivity of cancer cells to IR. To investigate the effect of ATR inhibitors on the G2/M checkpoint after irradiation in the background of ARID1A deficiency, the fraction of mitotic cells was measured by evaluating phosphorylated histone H3 (Ser-10). For this purpose, exemplarily two cell lines with ARID1A^-^ (SW48) and ARID1A^+^ (HCT116) were pre-treated for 1 h with the ATRi, VE822, were treated with 0.1 ug/mL nocodazole and irradiated with 4 Gy. Phospho-histone H3 staining at different times (4 h, 8 h, 24 h) thereafter was used to determine the mitotic index. The results clearly show that after exposure to IR, there is a clear reduction in the mitotic index at 4 h indicating the activation of the G2 checkpoint. At later times, cells recover from the G2 arrest. However, there was no apparent difference in the percentage of mitotic cells between VE822 treated and non-treated ARID1A^+^ CRC cell lines (**Figure 2B**). In contrast, ARID1A^-^ cells treated with VE822 showed a significantly (P<0.001) increased fraction of mitotic cells at 4 h, 8 h and 24 h after IR exposure. The results show that ATRi partially abrogates the G2-checkpoint in ARID1A^-^ (SW48) and ARID1A^+^ (HCT116) cells.

### Impact of ATRi and ARID1A deficiency on DSB repair

The molecular mechanism underlying the observed ATRi radiosensitization in ARID1A^-^ cells was evaluated by measuring RAD51 and γH2AX foci formation and decay after irradiation as markers for the engagement of HR and the overall repair, respectively. Radiation induced formation and decay of RAD51 and γH2AX foci were examined exemplarily in ARID1A^-^ (SW48) and ARID1A^+^ (HCT116) cells, specifically in the G2-phase of the cell cycle. For such analysis, cells were pulse labelled for 30 min with EdU, irradiated and fixed at different times thereafter. Repair foci were measured in EdU negative cells in the G2/M compartment as described in material and methods (**Supplemental Figure S8**).

**Figure 3:**
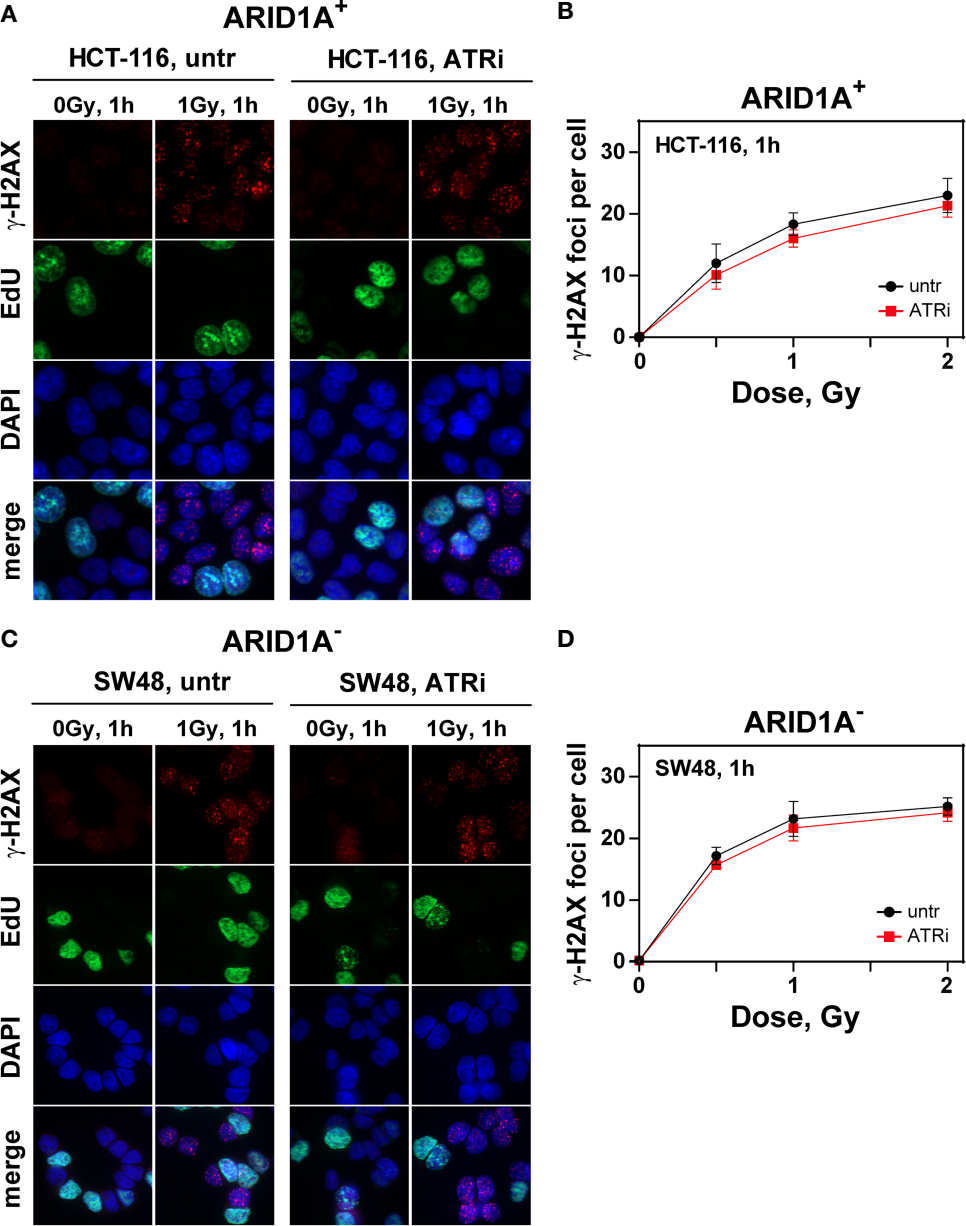
Effect of ATRi on ɣH2AX foci formation in G2-phase CRC cell lines.Maximum intensity projection (MIP) images of γH2AX foci (red) at tmax (1h) in G2-phase ARID1A^+^ **(A, B)** and ARID1A^-^ **(C, D)** cells without (untr) and with 20 nM VE822 (ATRi) in EdU^-^ (green) cells after exposure to the indicated IR doses. Cells were counterstained with DAPI (blue). The respective numbers of γH2AX foci at tmax as a function of IR dose are shown in **(B)** (ARID1A^+^ cells) and **(D)** (ARID1A^-^ cells). Results of 3 independent experiments are shown for CRC cell lines.

The number of radiation induced γH2AX foci as a measure for overall repair increases with time after irradiation and reaches its peak at about 1 h (tmax). The results showed that VE822 did not significantly affect initial γH2AX foci formation at tmax after 1 Gy, 2 Gy and 4 Gy in ARID1A proficient (HCT116, HCT15, Colo320DM) and ARID1A deficient (SW48, RKO) cell lines, (**Figure 3**; **Supplemental Figure S9**). However, in the ARID1A deficient cell line LS180, a slight but significant decrease in foci formation at tmax was observed (**Supplement Figure S9**).

In addition, the number of foci after irradiation with 0 Gy, 0.5 Gy, 1.0 Gy and 2 Gy was measured at different times after IR (1 h, 3 h, 6 h and 9 h) exemplarily in HCT116 and SW48 as a measure for repair of radiation induced DSBs. As shown in **Supplemental Figure S10**, there was no significant effect of ATRi on the kinetics of γH2AX foci formation and decay in both cell lines with or without ARID1A expression.

**Figure 4:**
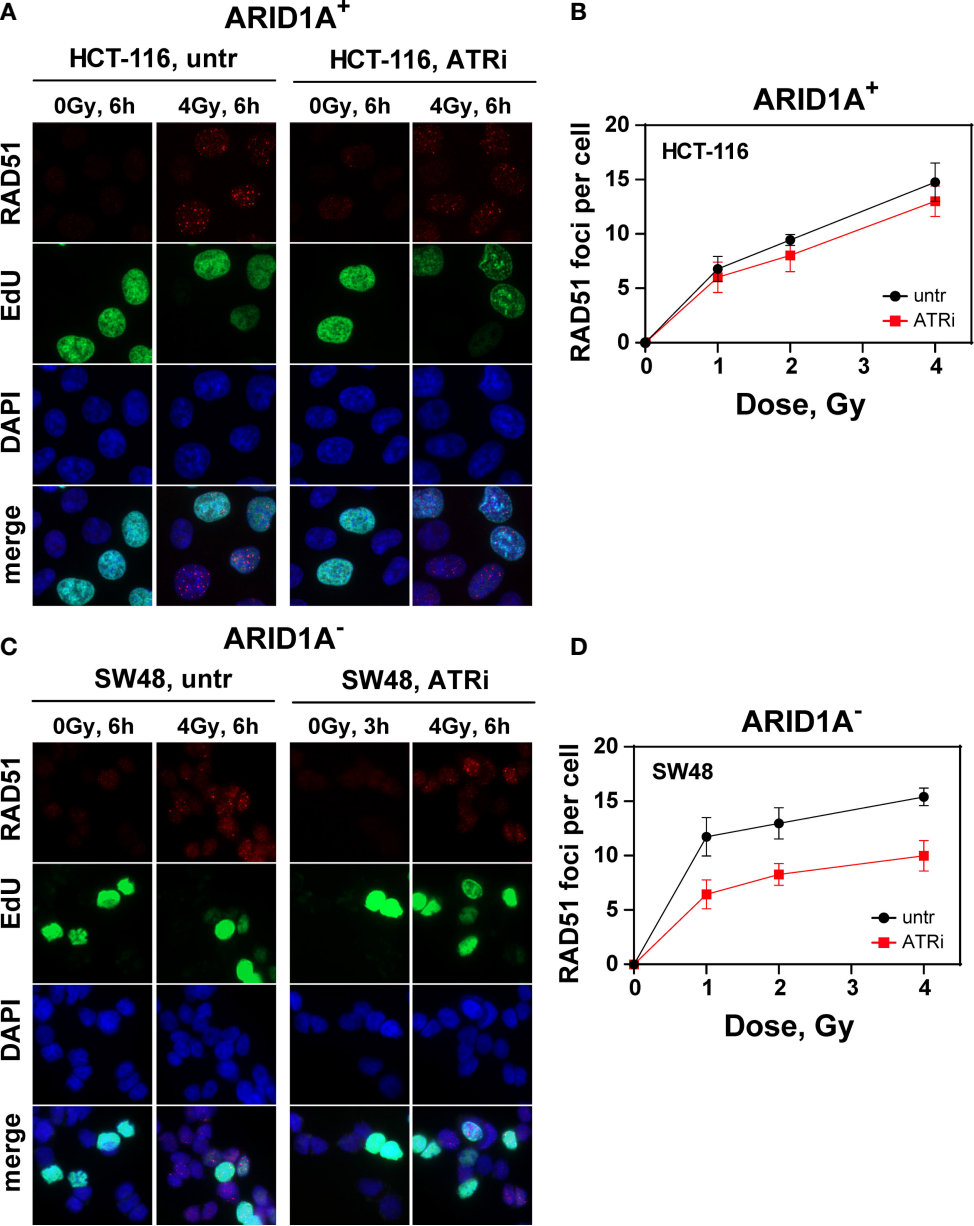
Effect of ATRi on RAD51 foci formation in G2-phase CRC cells. Maximum intensity projection (MIP) images of RAD51 foci (red) at tmax (6h) in G2-phase ARID1A+ **(A, B)** and ARID1A- **(C, D)** cells without (untr) and with 20 nM VE822 (ATRi) in EdU- (green) cells after exposure to the indicated IR doses. Cells were counterstained with DAPI (blue). The respective numbers of Rad51 foci at tmax as a function of IR dose are shown in **(B)** (ARID1A+ cells) and **(D)** (ARID1A- cells). Results of 3 independent experiments are shown for CRC cell lines.

In comparison, radiation induced RAD51 foci as a measure for HR reached its peak at about 6 h (t_max_) after irradiation. Irradiation with increasing radiation doses shows no significant difference between VE822 and sham treated ARID1A^+^ cell line HCT116 in the initial RAD51 foci formation at t_max_ (**Figures 4A, B**) and its decay with time (**Supplement Figure S11A**). However, VE822 significantly (p<0.01) decreased the initial number of IR induced RAD51 foci especially in the ARID1A^-^ cell line, SW48 (**Figures 4C, D** and **Supplemental Figure S11B**). **Figure 4D** clearly shows a significant (P <0.001) decrease of RAD51 foci in the presence of ATR inhibitor at t_max_ in ARID1A^-^ cell line SW48 (P<0.01). We also confirmed above results from other ARID1A^+^ (HCT15, Colo320DM) and ARID1A^-^ (RKO, LS180) CRC cell lines (**Supplement Figure S12**).

**Figure 5:**
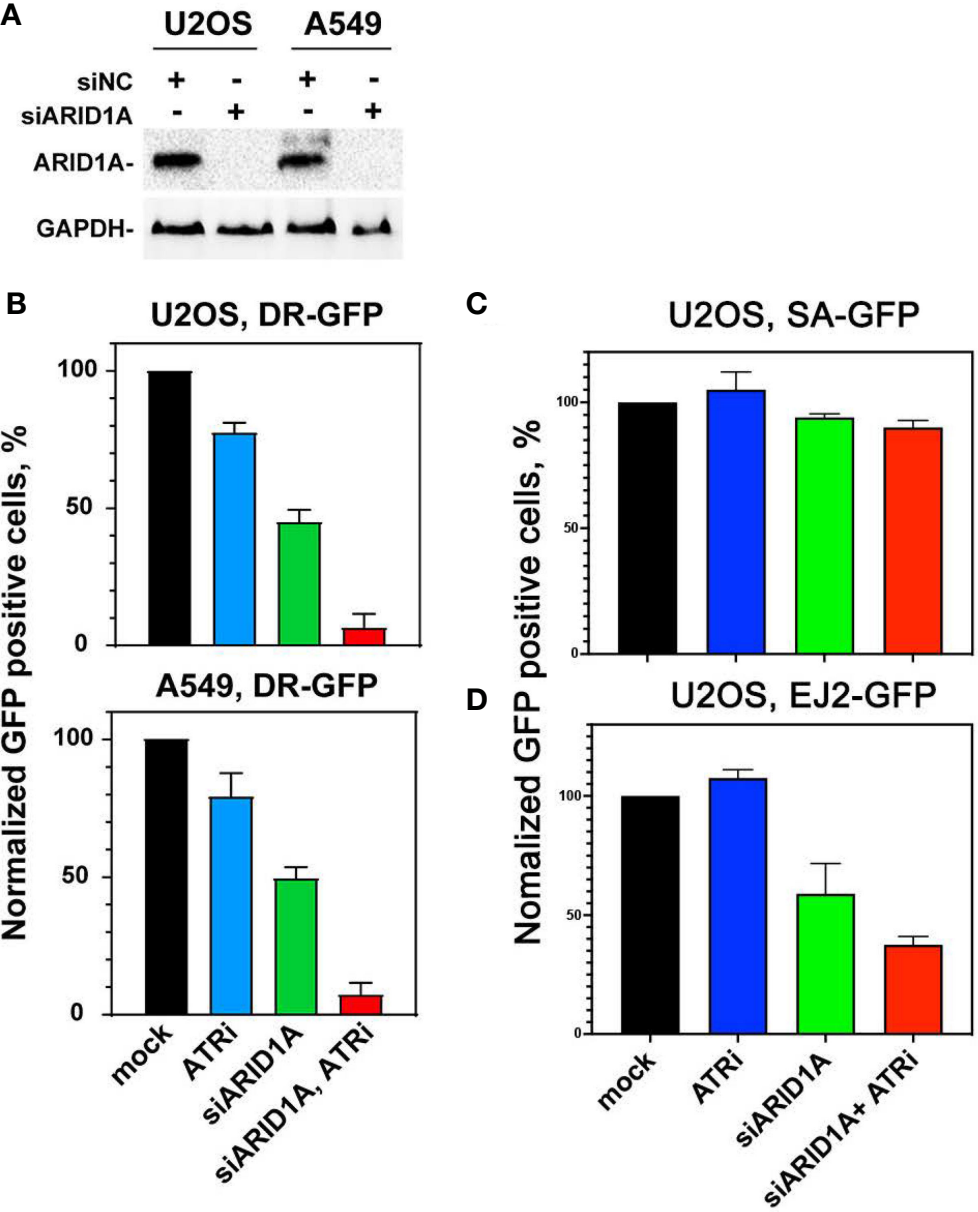
Effect of ATRi on HR repair in reporter cell lines. **(A)** Western blot results of ARID1A knock-down in DR-GFP-U2OS and DR-GFP-A549 reporter cells; GAPDH was used as an internal control. **(B)** Normalized GFP expression in DR-GFP- U2OS and DR-GFP-A549 reporter cells after treatment with control siRNA (mock), 20 nM VE822 (ATRi), ARID1A specific siRNA (siARID1A) and ATRi after knock-down of ARID1A (siARID1A, ATRi). **(C)** Normalized GFP expression in SA-GFP- U2OS reporter cells after treatment with control siRNA (mock), 20 nM VE822 (ATRi), ARID1A specific siRNA (siARID1A) and ATRi after knock-down of ARID1A (siARID1A, ATRi). **(D)** Normalized GFP expression in EJ2-GFP- U2OS reporter cells after treatment with control siRNA (mock), 20 nM VE822 (ATRi), ARID1A specific siRNA (siARID1A) and ATRi after knock-down of ARID1A (siARID1A, ATRi).Results of 3 independent experiments are shown for CRC cell lines.

To determine whether this effect on HR could be reproduced with an HR specific reporter assay, DR-GFP-U2OS and DR-GFP-A549 cell lines were used as a model system for HR repair. The reporter expresses GFP upon repair by HR of an I-SceI induced DSB, as outlined under materials and methods. At first, ARID1A expression was knocked-down in these 2 cell lines with siRNA (**Figure 5A**). Next, cells were treated with or without VE822. The data show, that ARID1A knock-down reduced the number of GFP^+^ cells and thus HR by 50.4 ± 8.3%. In comparison, ARID1A knock-down plus ATR inhibitor has the lowest fraction of GFP^+^ cells at 5.4 ± 1.7% (**Figure 5B**).

To further elucidate the impact of ATRi in ARID1A deficient cells on the single strand annealing repair pathway, SA-GFP-U2OS cells which report for single strand annealing (SSA) efficiency were used (21). After knock-down ARID1A expression (**Figure 5C**), SA-GFP-U2OS cells were treated or not withVE822. The results showed that ARID1A knock-down (94 ± 3.3%) and ARID1A knock-down plus ATR inhibitor (88 ± 4.1%) did not significantly reduce SSA (**Figure 5C**).

To test if ARID1A knock-down affects altNHEJ, we used the well-established EJ2-GFP reporter for alt NHEJ (22). The results show that ARID1A knock-down impaired altNHEJ (65 ± 9.3%). In addition, ARID1A knock-down plus ATR inhibitor was 40 ± 5.3% (**Figure 5D**). We also tested different concentrations of VE822 in the DR-GFP reporter cell line with or without ARID1A knock-down. The results show (**Supplement Figure S13**) that treatment with 20 nM VE822 of DR-GFP-U2OS reporter cell line following ARID1A knock-down halved HR repair (45.1 ± 9.1%). This result confirmed that VE822 could significantly potentiate the effect on HR after knock-down of ARID1A.

### ATRi sensitizes primary tumors of CRC patients with ARID1A deficiency

**Figure 6:**
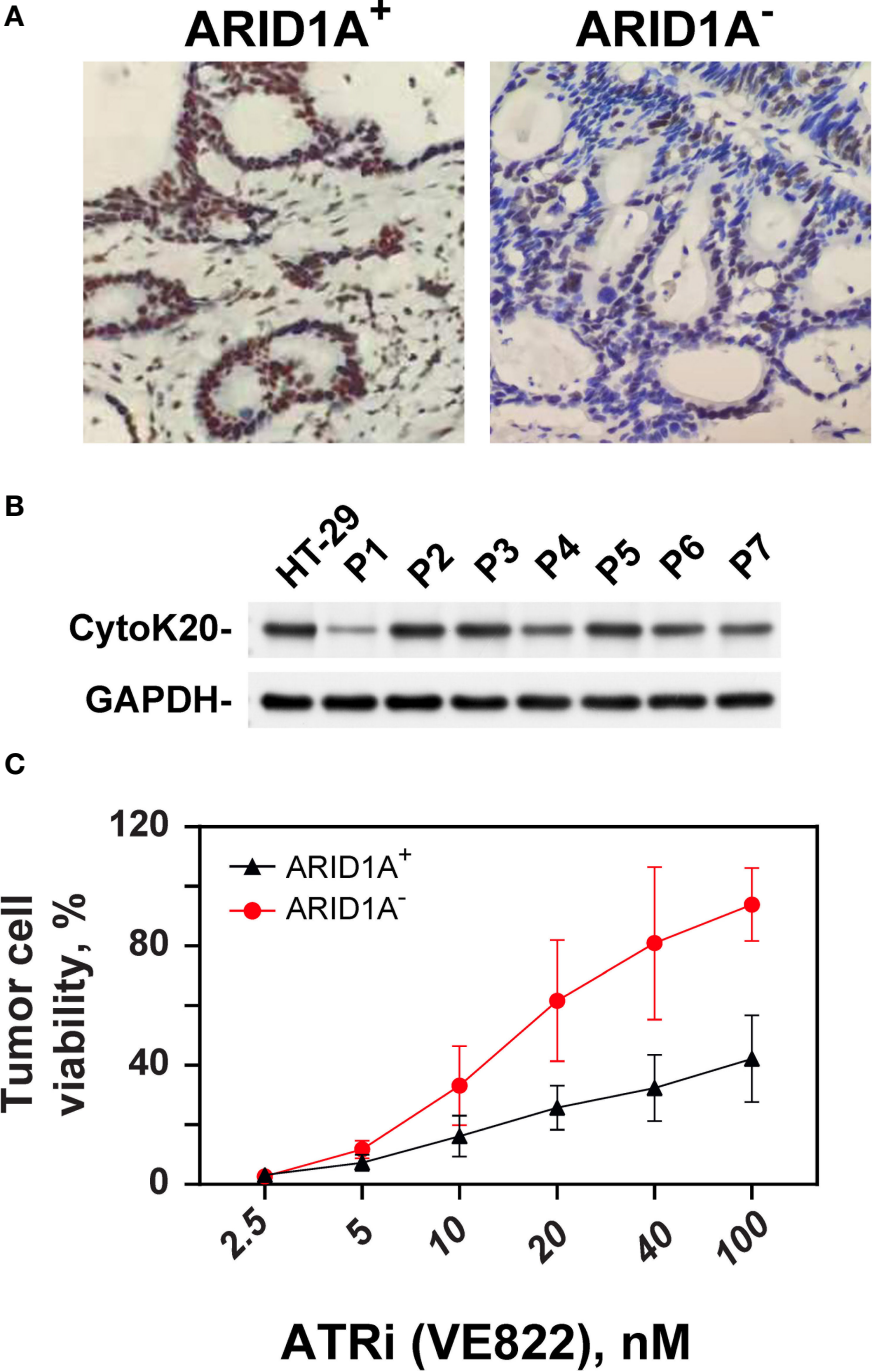
Effect of ATRi on ex vivo explants from CRC patients. **(A)** Example of IHC staining of ARID1A expression from clinical CRC tumor cells with and without ARID1A expression. **(B)** Western blot of CK20 expression from primary CRC cells; GAPDH was used as an internal control. **(C)** ATP-Tumor Chemosensitivity Assay for the effect of the ATR inhibitor VE822 on *ex-vivo* cells from CRC patients. ATP activity was measured after treatment of *ex-vivo* cells with concentration ranged from 0, 2.5 up to 100 nM in cells from CRC patients with (+) and without (-) ARID1A expression.

Tumor specimens from a total of 46 CRC patients were evaluated with respect to ARID1A expression. Five of 46 tumors (10.9%) showed reduced ARID1A expression in tumor cell nuclei (**Figure 6A**). Patients’ clinicopathologic characteristics are summarized in **Table 2**. Loss of ARID1A expression was not associated with gender, age, tumor location, TNM stage, or tumor size. However, there is a statistically significant difference between ARID1A expression and pathologic differentiation, as well as lymphatic penetration (P<0.05).

**Table 2:** Clinicopathologic characteristics and ARID1A expression in patient samples of colorectal cancer.

Characteristics	ARID1A Expression		
	Negative (n=5)	Positive (n=41)	P
**Age**			>0.05
≤50	3	18	
>50	2	23	
**Gender**			>0.05
Male	3	34	
Female	2	7	
**Tumor location**			>0.05
Right Colon	2	10	
Left colon	0	2	
Rectal	3	29	
**TNM stage (AJCC)**			>0.05
I	0	14	
II	1	10	
III	4	17	
**Pathologic differentiation**			<0.05
Poor	3	2	
Moderate	2	10	
Well	0	29	
**Tumor size (cm)**			>0.05
≤5	1	9	
>5	4	32	
**Lymphatic penetration**			<0.05
Negative	2	36	
Positive	3	5	

AJCC, American Joint Committee on Cancer; TNM, Tumor-node- metastasis; ARID1A, AT-rich interactive domain 1A. The correlation between ARID1A expression and clinicopathological characteristics was analyzed by χ 2 test, or Kruskal-Wallis H test, depending on the data analyzed.

The sensitizing effect of VE822 on colorectal cancer with and without ARID1A expression was evaluated in an *ex vivo* experimental setting. Cells from primary tumors with and without ARID1A expression were tested in an ATP-based tumor chemosensitivity assay (ATP-TCA). Of the 46 colorectal cancer specimens, 43 cases completed the ATP-TCA test, and 3 specimens were discarded due to low cell number or weak colon cancer markers CK20 (**Figure 6B**). The evaluable rate of the ATP-TCA method for colorectal cancer specimens was 93.4%. All 43 cases (4 negative colorectal cancer specimens and 39 positive colorectal cancer specimens) were used to generate a dose-response curve of VE822 using the ATP-TCA method (**Figure 6C**, better cell viability on y axis). The results clearly demonstrate that the ARID1A negative group is significantly (p <0.001) more sensitive to VE822 compared with ARID1A positive group, with an IC50 value of 98.8 ± 45.4 nM and 19.83 ± 7.83 nM for the ARID1A positive and negative group, respectively. The IC50 values of VE822 in both ARID1A expression groups of primary CRC cancers is close to the IC50 values of in the *in vitro* colony formation assay of ARID1A^-^ and ARID1A^+^ cell lines.

## Discussion

Identification of cancer specific vulnerability arising from mutations in the context of the SWI/SNF chromatin remodeling complex has high clinical significance, because it paves the way to the development of more effective treatments combining radiotherapy with chromatin-targeted therapy options (23). Among the SWI/SNF complex subunits, ARID1A has the highest frequency of mutation in cancers and is related to poor prognosis and high tumor activity (24). It is also engaged in DNA repair, a molecular process that plays a significant role in the resistance of tumor cells to radiation and chemotherapy (16). Thus, efforts have been made to develop cancer therapeutics exploiting the mutational status of ARID1A in cancer patients (10, 25). Currently, research related to ARID1A deficiency in cancer has led to the identification of DSB repair pathways that are compromised, increasing thus the vulnerability of cancer cell lines to the applied treatment modalities (26, 27). Many of these treatments rely on the idea of synthetic lethality, in which only the simultaneous perturbation of two genes results in cellular or organismal death in normal cells, but a single perturbation is sufficient to kill a cell with perturbation (mutation) in one of these genes (28). According to our previous work (14), ARID1B knock-down did not greatly influence proliferation or plating efficiency, while it significantly radiosensitized ARID1A^-^ cells. As pointed out by Shen et al. (16), ARID1A plays a role in regulating the DNA damage checkpoint activated by DSBs and loss of ARID1A function leads to increased reliance on ATR (17). Thus, ATR inhibitors have been explored as a synthetic lethal strategy for treating patients with ARID1A^-^ tumors.

Like other epigenetic complexes, paralog pairs of the SWI/SNF complex such as ARID1A/ARIDB (14) and SMARCA2/SMARCA4 (29), are recognized as vulnerabilities in cancer. In the present study, we corroborate our previous finding that knock-down of ARID1B sensitizes specifically ARID1A^-^ cells. According to Helming et al. (30), ARID1B is not only the closely connected paralog of ARID1A, but also a synthetic target in ARID1A^-^ cells. However, the mechanistic basis of synthetic lethality between ARID1A and ARID1B was unclear. Our previous work provided one possible mechanism as to how depletion of ARID1B in ARID1A^-^ CRC cell lines could sensitize cells to treatments: by reducing HR (14). Nonetheless, there are challenges in the design of an ARID1B-targeted therapy. Specifically, ARID1B lacks a small molecule-binding enzymatic domain, so that the targeting of a protein-protein or protein-DNA interaction is necessary (31). Encouragingly, several studies have shown that ARID1A directed synthetic lethality can be attained through diverse molecular mechanisms (32). ATR inhibitors were shown to be a synthetic lethal partner of ARID1A deficiency (16, 17). ATR is a key element in the cellular DDR and is activated by single-stranded DNA regions generated during replicative stress or resection dependent DSB processing. Deficiency of ARID1A is accompanied by defects in cell cycle progression and topoisomerase 2A (TOP2A), both of which increase the dependence on ATR checkpoint activities (17). Suppression of ATR induces genomic instability and thus activation of programmed cell death in ARID1A^-^ cancer cells (17). Hence, ATR inhibitors are likely to present a synthetic lethal strategy to target cancer cells with mutant or low ARID1A expression.

The present data show that combining ionizing radiation with ATR inhibitors is highly effective in eradicating ARID1A^-^ CRC cancer cells. Both ATR inhibitors (VE821 and VE822) could significantly increase radiation sensitivity in ARID1A^-^ CRC cells, while at the concentrations used, no sensitizing effect was evident for CRC lines with ARID1A^+^. Of particular importance, ATR inhibitor plus siRNA-mediated ARID1B knock-down further increases the radiosensitivity of ARID1A^-^ CRC cells.

Therefore, our data suggest that ATRi has a potential synthetic lethality effect with respect to the radiation sensitivity of ARID1A^-^ CRC cells. This effect tends to be higher in S-phase enriched cell populations, indicating a higher sensitizing effect on cells with higher proliferation capacity, i.e. in tumor cells compared to normal cells, which should increase the therapeutic window. The rather marginal increase in the radiation sensitivity of cells in the middle S phase compared to cells in the early S could possibly be increased by comparing cells in the early G1 phase versus middle S phase.

In order to further investigate the molecular mechanism of radiosensitization, the effect of ATRi on the formation of DSB repair foci in ARID1A^-^ and ARID1A^+^ CRC cell lines was explored. RAD51 foci formation, as a marker for HR and γ-H2AX as a marker of DSBs and overall repair were examined in G2/M phase (Edu^-^) cells at different times after exposure to different doses of IR. ATRi did not increase γH2AX foci in ARID1A^-^ or in ARID1A^+^ CRC cell lines, suggesting that ATRi has only a small effect on overall DSB repair. The concentration of 20 nM of ATRi employed is relatively low and thus, an effect at higher inhibitor concentrations cannot be excluded. In contrast, ATRi significantly decreased Rad51 foci formation in ARID1A^-^ CRC cells, but not in ARID1A^+^ cell lines, suggesting that ATRi mediated radiosensitization was associated mainly with inhibition of HR.

In order to strengthen the results on HR repair, the well-established DR-GFP reporter assay (33) was used to specifically measure the effect of ARID1A deficiency on HR repair. The results showed that knock-down of ARID1A resulted in reduced HR repair. Shen et al. (16) suggest that ARID1A recruits BAF to DSBs through interactions with ATR and promotes DSB end-resection. BAF complexes have been implicated in DSB repair by regulating DSB end-resection and Rad51 loading. The consequences of ARID1A deficiency in this setting is a homologous recombination defect generating sensitivity to ATR inhibitors. The observation that ATRi did not equally reduce the different repair pathways that play a role in S/G2 phases of the cell cycle and where end-resection is implicated in the repair pathways (HR and SSA), may either derive from the concentration used or the duration of the treatment with ATRi. It has been shown that distinct modes of ATRi treatment, i.e. acute and chronic treatment, lead to different outcomes as to how DNA lesions are repaired. Acute VE-821 treatment of U2OS cells was shown to impair PARPi-induced RAD51 foci, as a measure for HR, but did not alter RAD52 foci, as a measure for SSA (34)

In addition, Tsai et al. (35) have shown that ARID1A deletion increases transcription-replication conflicts and R-loop associated genome instability. Dysregulation of replication and transcriptional programs is associated with altered targeting of TOP2A to R-loop prone regions (35). Taken together, evidence exists from different studies that HR repair can be affected by ARID1A knock-down, especially following exposure to an ATR inhibitor (16, 35).

Multiple studies have indicated that ATR and ARID1A could regulate cell cycle progression (17, 36). Our study shows that ARID1A^-^ cells treated with ATRi showed significant increase in the numbers of mitotic cells after IR exposure, i.e. release from radiation induced G2 arrest. Williamson et al. (17) suggested that loss of ARID1A caused a reduced rate of S-phase progression and increased utilization of the G2/M checkpoint. In a review from Caumanns et al. (32), it was demonstrated that ARID1A loss in HCT116 cells resulted in the accumulation of cells in G2/M phase of the cell cycle. Our results further revealed that ATR inhibition forces ARID1A^-^ cells in G2 phase into M phase with DNA damage that severely compromises genomic instability. In addition, we found out that ATRi decreased HR, as measured by Rad51 foci formation and thus increased the fraction of non-repaired DSBs, as well as of DSBs processed by error-prone repair pathways, thereby sensitizing ARID1A^-^ CRC cells to IR.

There is evidence that ATR inhibitors are efficient in the treatment of ARID1A^-^ tumor cells, however clinical verification of the concept is still lacking. Interim results of a phase II study of the ATR inhibitor ceralasertib in ARID1A-deficient and ARID1A-intact advanced solid tumor malignancies with an objective response rate (OR) of 20% in patients with ARID1A deficiency, compared to 0% OR in patients with wild type ARID1A, have been recently presented (37). Thus clinical trials on combined effect of ATRi and radiotherapy or chemotherapy are urgently needed and are indeed emerging (38, 39) This led us to investigate the efficacy of ATR inhibitors in an *ex-vivo* system as a translational stepping-stone by using primary tumor specimens from surgical explants of CRC patients with and without ARID1A expression. The results show that in 5 of 46 cases of colorectal cancer (10.9%) no ARID1A protein expression was detectable. We did not find any significant correlation between loss of ARID1A negative expression and gender, age, tumor location, TNM stage, or tumor size. However, there is a statistically significant association between ARID1A negative expression and poor pathologic differentiation and lymphatic penetration. These data, suggest that ARID1A may play an important role in the progression and invasion of CRC cells. Lee, et al. (40) also showed that ARID1A loss is associated with poor tumor differentiation, lymphovascular invasion and microsatellite instability. Wei, et al. (19) report that ARID1A protein expression was a prognostic marker for better OS in stage IV CRC. Collectively, these evidences show that ARID1A mutations may be candidate predictors of new targeted therapeutic approaches for CRC patients.

The ATP-based tumor chemosensitivity assay (ATP–TCA) was developed in the early 1990s, and has proved to be a useful tool for cell-based research and drug development work (41). The ATP-TCA assay is preferable to previous comparable methods in terms of standardization, evaluability, tumor cell number required, reproducibility and accuracy (42). It is possible to test cells from needle biopsies and malignant effusions, as well as solid tumor biopsies. In our study, *ex-vivo* material from a total of 43 untreated colon cancer patients underwent ATP-TCA directed ATRi therapy. The ATP-TCA assay shows that the ATR inhibitor VE-822 has tumor suppression rates by a factor of about 5 higher in ARID1A negative compared to ARID1A positive CRC cells. These data on the effect of ATRi on primary tumor cells from surgical material strongly support the translational research relevance and the potential clinical application of the ARID1A/ATRi concept.

In conclusion, the present study shows for the first time the impact of ATRi on ARID1A mutation in CRC cells, as well as in primary tumor material from CRC patients. ATRi are candidates to selectively increase the effectiveness of radiation chemotherapy in ARID1A deficient rectal carcinoma cells in the clinic, as small to moderate increase in cell death after conventional radiation dose fraction of 1.8 Gy can be potentiated during the fractionated application of IR. It was shown that ARID1A deficiency suppresses HR, which was suggested to be one of the mechanisms for the observed increase in radiation sensitivity upon ATR inhibition. Since homologous recombination is predominantly effective in S- and G2-phase, i.e. in proliferating cells, it is likely that normal tissue will not be sensitized to the extent of tumor cells.

Collectively, our results provide mechanistic insights into how ATR inhibitors cause radiosensitization in ARID1A defective CRC cells and point to new therapeutic avenues for patients with ARID1A deficient tumors.

## Data availability statement

The original contributions presented in the study are included in the article/**Supplementary Material**. Further inquiries can be directed to the corresponding authors.

## Ethics statement

The studies involving human participants were reviewed and approved by the independent ethics committee of the MianYang Fulin hospital. The patients/participants provided their written informed consent to participate in this study.

## Author contributions

MS directed the study. SX, AS, YE, EM, and MK designed and performed experiments. MG performed experiments. SX, AS, GI, EM, and MS analyzed and interpreted the data. SX and AS wrote the manuscript. All authors contributed to the article and approved the submitted version.

## Funding

This work is funded by the Deutsche Forschungsgemeinschaft (DFG) as part of the Graduate School (GRK1739/3).

## Acknowledgments

We acknowledge support by the Open Access Publication Fund of the University of Duisburg-Essen.

## Conflict of interest

The authors declare that the research was conducted in the absence of any commercial or financial relationships that could be construed as a potential conflict of interest.

## Publisher’s note

All claims expressed in this article are solely those of the authors and do not necessarily represent those of their affiliated organizations, or those of the publisher, the editors and the reviewers. Any product that may be evaluated in this article, or claim that may be made by its manufacturer, is not guaranteed or endorsed by the publisher.
