# Supplementary material for: Selective vulnerability of ARID1A deficient colon cancer cells to combined radiation and ATR-inhibitor therapy
Source: Front Oncol. 2022 Sep 30;12:999626. doi: 10.3389/fonc.2022.999626 (PMC9561551; doi:10.3389/fonc.2022.999626)
Supplement: Supplementary file 1 [file DataSheet_1.docx]

**Supplementary Materials**

**Table S1:** IC50 values of ATR inhibitors in ARID1A^+^ and ARID1A^-^ colon cancer cell lines.

|  | ARID1A + | | | ARID1A - | | | P^1^ |
| --- | --- | --- | --- | --- | --- | --- | --- |
|  | HCT15 | HCT116 | Colo320DM | SW48 | RKO | LS180 |  |
| VE822 (nM) | 88.95±22.3 | 76.76±16.7 | 99.54±18.3 | 19±1.5 | 21±1.3 | 20±2.3 | <0.001 |
| VE821 (µM) | 6.3±1.3 | 5.75±2.2 | 5.3±1.7 | 1.1±0.3 | 1.3±0.18 | 1.4±0.3 | <0.001 |

P^1^: ANOVA was used to calculate the statistical significance between groups.

**Table S2:** The radiosensitivity of ARID1B knock down and ATR inhibitors on ARID1A^+^ and ARID1A^-^ colon cancer cell lines

|  | ARID1A + | | | ARID1A - | | |  |  |
| --- | --- | --- | --- | --- | --- | --- | --- | --- |
|  | HCT15 | HCT116 | Colo320DM | RKO | SW48 | LS180 | P^1^ | P^21^ |
| Control SF2 | 0.39 ± 0.019 | 0.38 ± 0.021 | 0.4±0.042 | 0.36 ± 0.018 | 0.35 ± 0.019 | 0.35 ± 0.058 | >0.05 |  |
| VE821 (1 μM) SF2 | 0.35 ± 0.012 | 0.34 ± 0.023 | 0.38 ± 0.021 | 0.28 ± 0.032 | 0.20 ± 0.045 | 0.28 ± 0.033 | <0.01 | <0.05 |
| VE822 (20 nM) SF2 | 0.34 ± 0.055 | 0.35 ± 0.031 | 0.37 ± 0.018 | 0.25 ± 0.017 | 0.13 ± 0.016 | 0.22 ± 0.011 | <0.001 | <0.001 |
| ARID1B KD SF2 | 0.36 ± 0.027 | 0.36 ± 0.03 | 0.37 ± 0.045 | 0.30 ± 0.016 | 0.26 ± 0.013 | 0.30 ± 0.011 | <0.05 |  |
| VE821 (1 μM) +  ARID1B KD SF2 | 0.33 ± 0.032 | 0.35 ± 0.012 | - | 0.21 ± 0.039 | 0.14 ± 0.041 | - | <0.001 |  |
| VE822 (20 nM) + ARID1B KD SF2 | 0.33 ± 0.078 | 0.34 ± 0.051 | - | 0.16 ± 0.047 | 0.10 ± 0.018 | - | <0.001 |  |

SF2: survival fraction in 2Gy. P^1^: ANOVA was used to calculate the statistical significance between ARID1A^+^ and ARID1A^-^groups; P^2^: ANOVA was used to calculate the statistical significance between ATRi and ARID1B KD in ARID1A^-^groups.

**Table S3:** The radiosensitising effect of ATRi in ARID1A mt colon cancer cell lines in early S and S phase quantified by the dose modifying factor (DMF)

| Cell line | Cell cycle phase | Dose modifying factor (DM) due to ATRi | P^1^-value  for the ATRi effect | P^1^ for dependence of DM on cell cycle position |
| --- | --- | --- | --- | --- |
| HCT15 | Early S phase | 0.969 (95% CI:0.891-1.055) | 0.44 | 0.88 |
| HCT15 | Mid S phase | 0.968 (95% CI:0.928-1.010) | 0.12 |  |
| HCT116 | Early S phase | 0.988 (95% CI:0.903-1.081) | 0.78 | 0.29 |
| HCT116 | Mid S phase | 0.976 (95% CI:0.863-1.104) | 0.68 |  |
| SW48 | Early S phase | 0.828 (95% CI:0.770-0.891) | <0.0001 | 0.09 |
| SW48 | Mid S phase | 0.740 (95% CI:0.660-0.830) | <0.0001 |  |
| RKO | Early S phase | 0.827 (95% CI:0.765-0.894) | 0.0002 | 0.44 |
| RKO | Mid S phase | 0.780 (95% CI:0.736-0.826) | <0.0001 |  |

^1^P: Colony data were analyzed using a linear-quadratic model describing the dependence of the logarithm of cellular survival. ANOVA was used to calculate the statistical significance between groups.


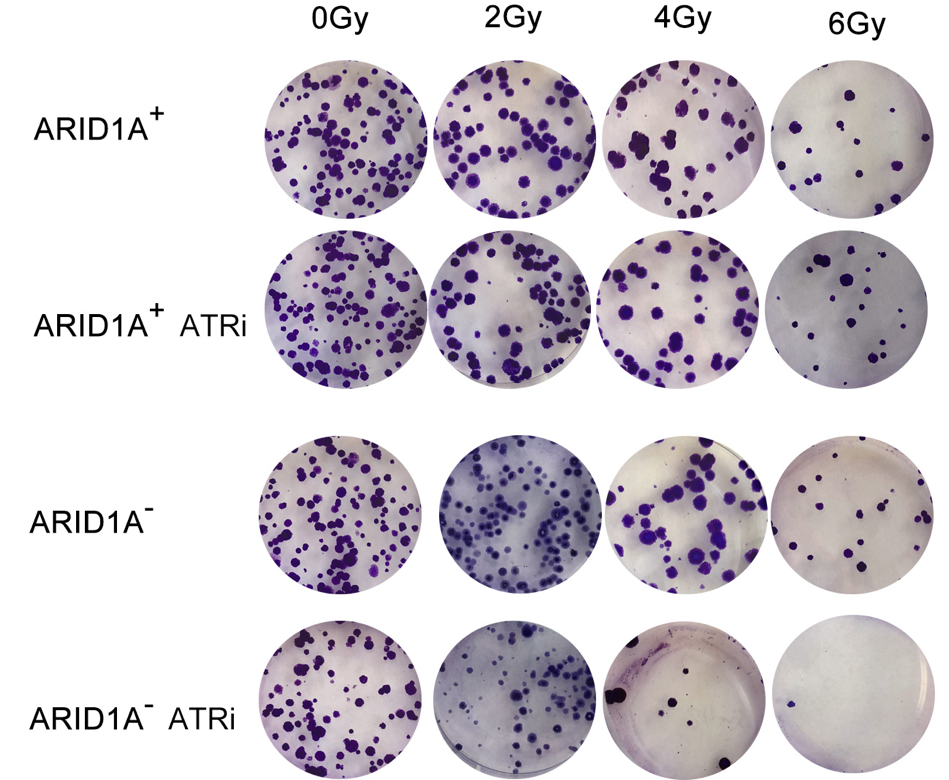


**Figure S1: Colony Forming Assay results of ATRi (VE822) on radiosensitivity.**

ARID1A+ and ARID1A- cell lines were pre-treated for 1 h with 20 nM VE822 and irradiated with 0 Gy, 2 Gy, 4 Gy and 6 Gy.


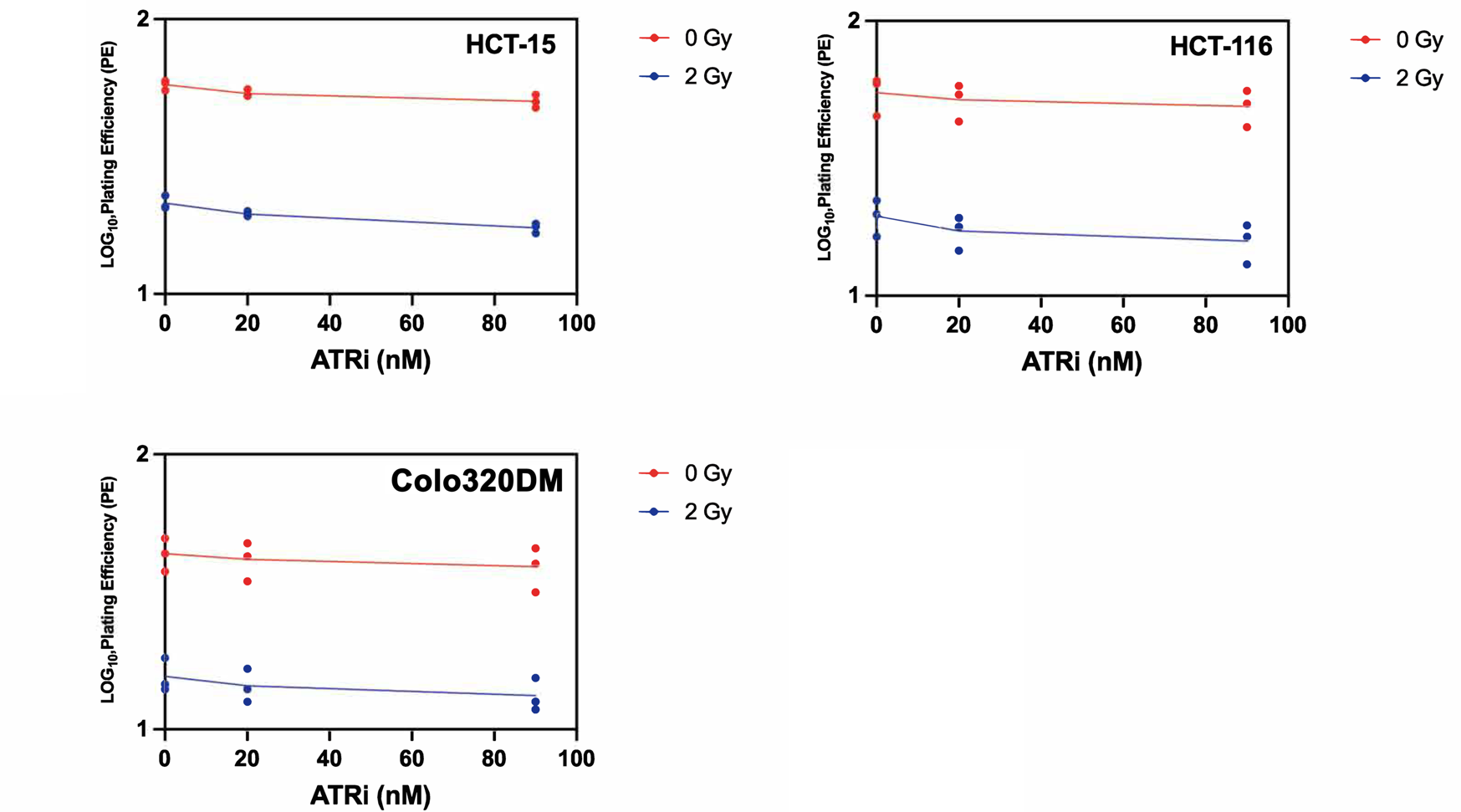


**Figure S2: Effect of ATR inhibitor (VE822) in ARID1A^+^ cell.**

Plating efficiency of sham treated (untr) and different concentration of VE822 treated (ATRi) cells were plotted as log10 for ARID1A^+^ cell lines. Results of 3 independent experiments are shown for CRC cell lines.





**Figure S3: Effect of ARID1A knockdown in ARID1A^+^ cell plus ATR inhibitor (VE822) on radiosensitivity**. A: Western blot results of ARID1A knockdown by siRNA. B: The effect of ARID1A knockdown in ARID1A^+^ CRC cell lines and the effect of ARID1A knockdown plus ATR inhibitor (VE822) in ARID1A^+^ CRC cell lines. P values indicate the results from the ANOVA F-test. Results of 3 independent experiments are shown for CRC cell lines (M±SD).


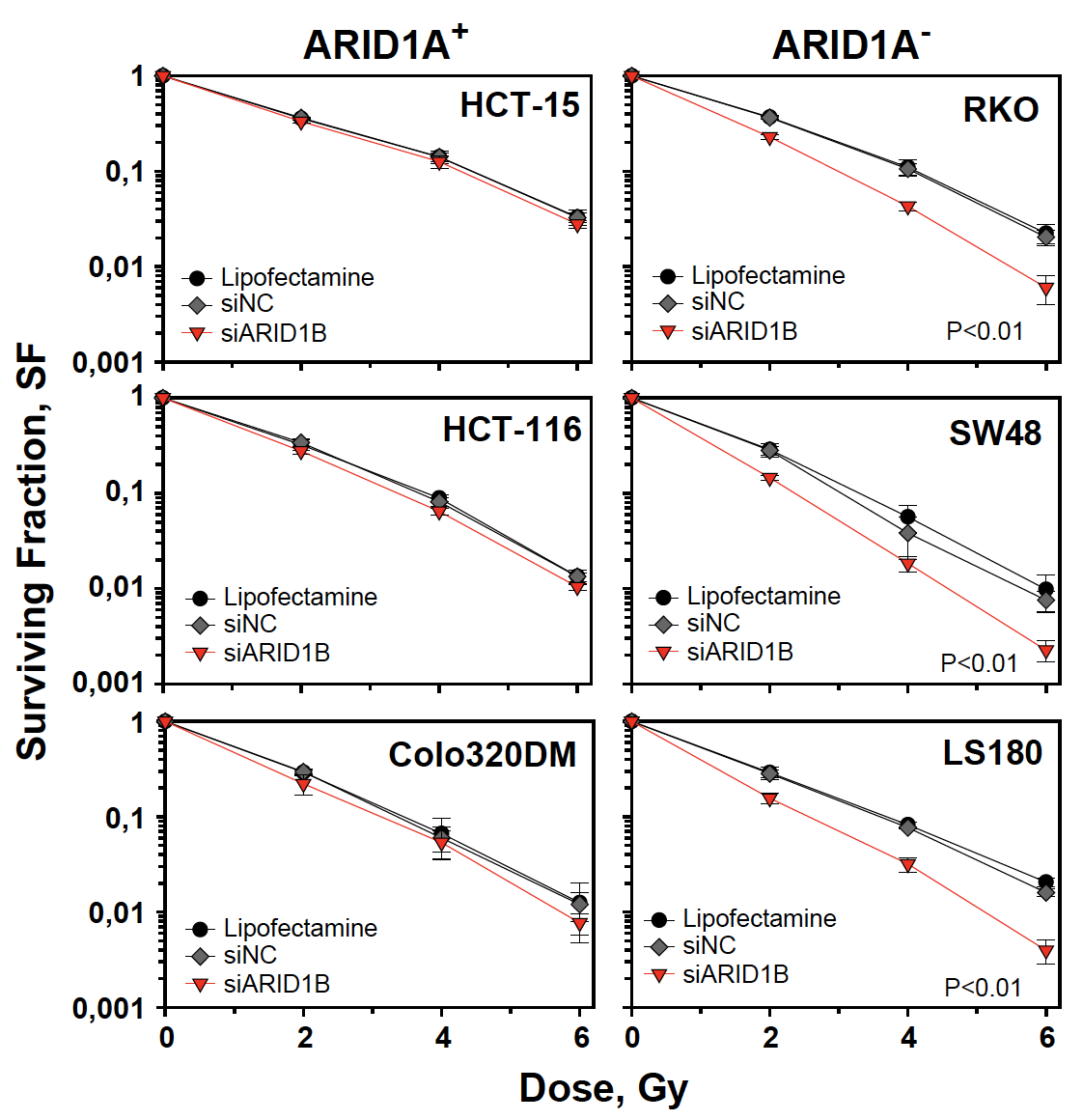


**Figure S4: Effect of ARID1B/ARID1A on radiosensitivity.**

Survival curves for ARID1B depleted and non-targeted siControl transfected cells are shown together with residual values of the observed surviving fractions. Surviving fraction: Natural logarithm of the surviving fraction normalized to the mean of the sham irradiated controls, exposed to control- or ARIDIB-siRNA. P values indicate the results from the ANOVA F-test for the radiation response-modifying effect ofARID1B-knockdown. Results of 5 independent experiments are shown for CRC cell lines (M±SD).

**

**

**Figure S5: Effect of ATRi/ARID1A plus ARID1B knock down on radiosensitivity.**

A:VE821 plus ARID1B knock down; B: VE822 plus ARID1B knock down. P values indicate the results from the ANOVA F-test for the radiation response-modifying effect of ARID1B-knockdown. Results of 3 independent experiments are shown for CRC cell lines (M±SD).


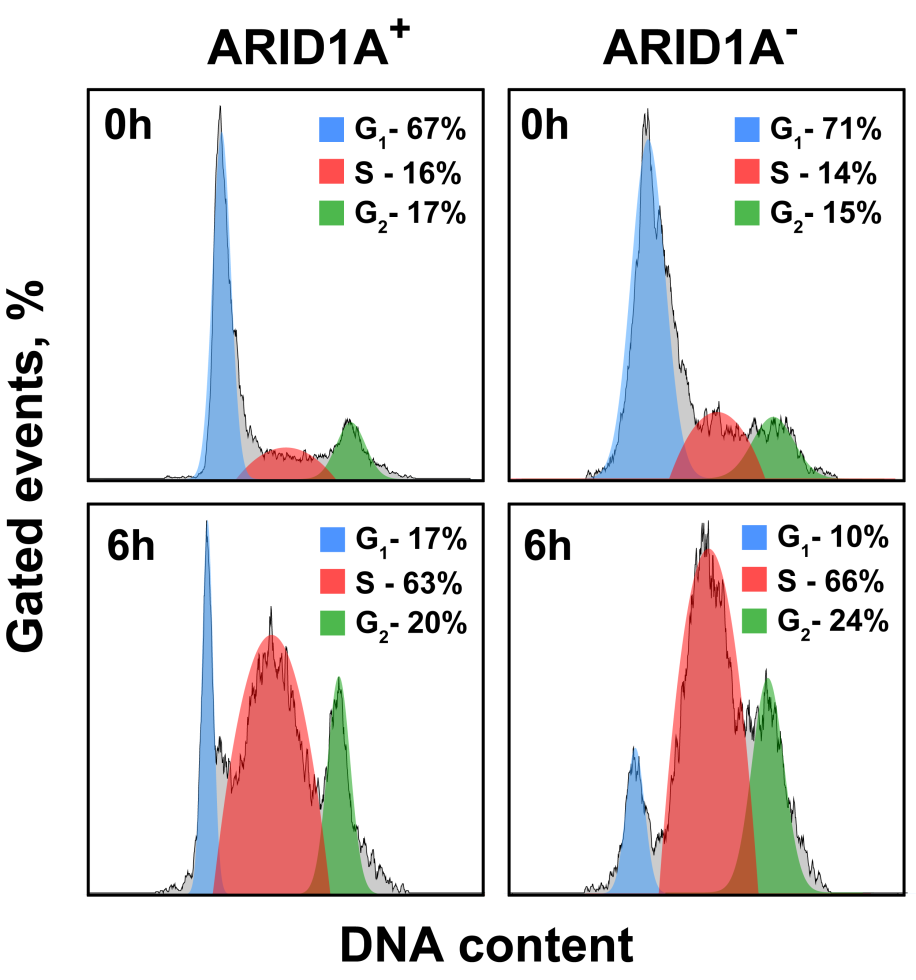


**Figure S6: Cell cycle synchronization by aphdicoline.**

ARID1A^+^ and ARID1A^-^ colon cancer cells were synchronized by 20 h incubation with aphidicolin. After aphidicolin removal, cells were collected at different time thereafter (from 0 to 24 h). DNA content was measured by flow cytometry by tracing the fluorescence intensity of the cells. Treatment with aphidicolin for 20 h synchronizes cells at early S-phase stage of the cell cycle and at mid S-phase at 6 h after release from the aphidicolin block.


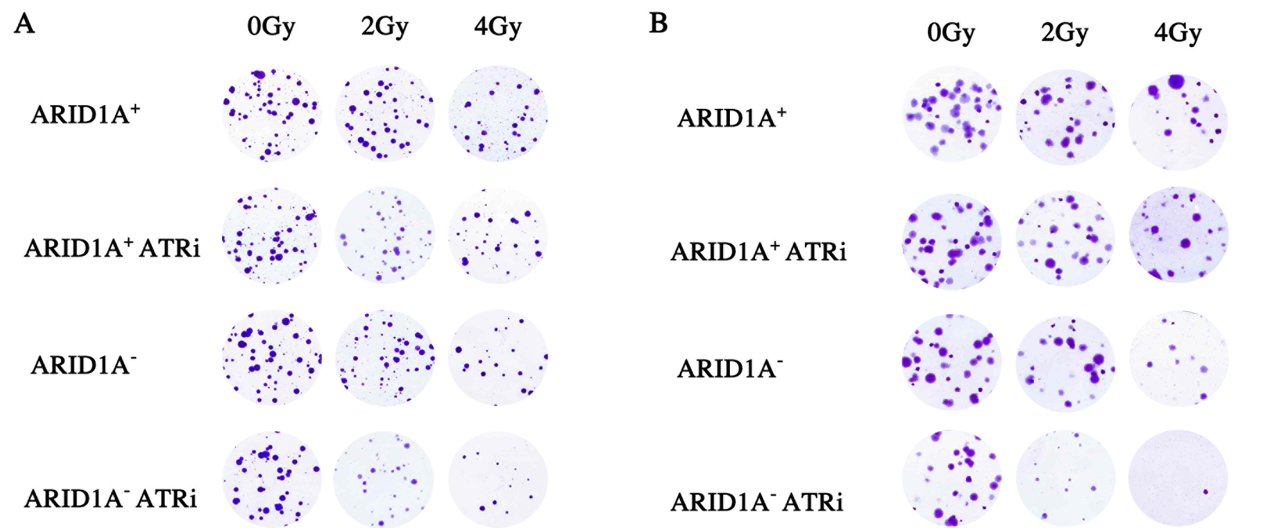


**Figure S7: Colony Forming Assay results of Cell cycle effect on ATRi/ARID1A.**

**A:** Synchronized cells in early S were pre-treated for 1 h with VE822 and irradiated thereafter with 0 Gy, 2 Gy and 4 Gy. **B:** Synchronized cells in mid S phase were pre-treated for 1 h with VE822 and irradiated thereafter with 0 Gy, 2 Gy and 4 Gy.

**
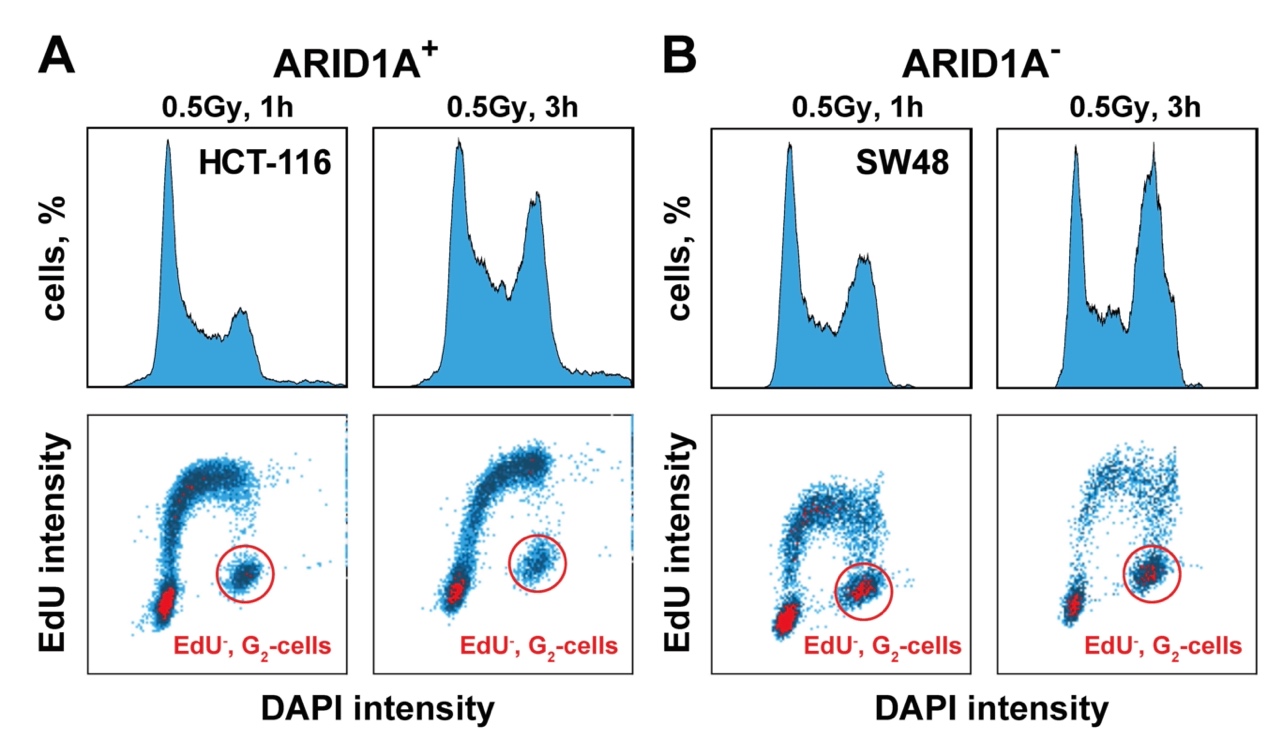
**

**Figure S8: Foci formation in the G2-phase based on EdU staining.**

A: ARID1A^+^ CRC cells EdU staining; B: ARID1A^-^ CRC cells. Histogram and dot plots of data obtained by quantitative image-based cytometry analysis (QIBC) of ARID1A^+^ and ARID1A^-^ CRC cells. The gates used to score γ-H2AX or RAD51 foci in a cell cycle specific manner are also indicated. For the current analysis EdU-, G2-cells were selected.





**Figure S9: Effect of ATRi on ɣH2AX foci formation in G2-phase CRC cell lines.**

The respective numbers of ɣH2AX foci at tmax (1h) in G2-phase ARID1A^+^ and ARID1A^-^ cells without (untr) and with 20 nM VE822 (ATRi) after exposure to the indicated IR doses. Results of 3 independent experiments are shown for CRC cell lines(n=3)..


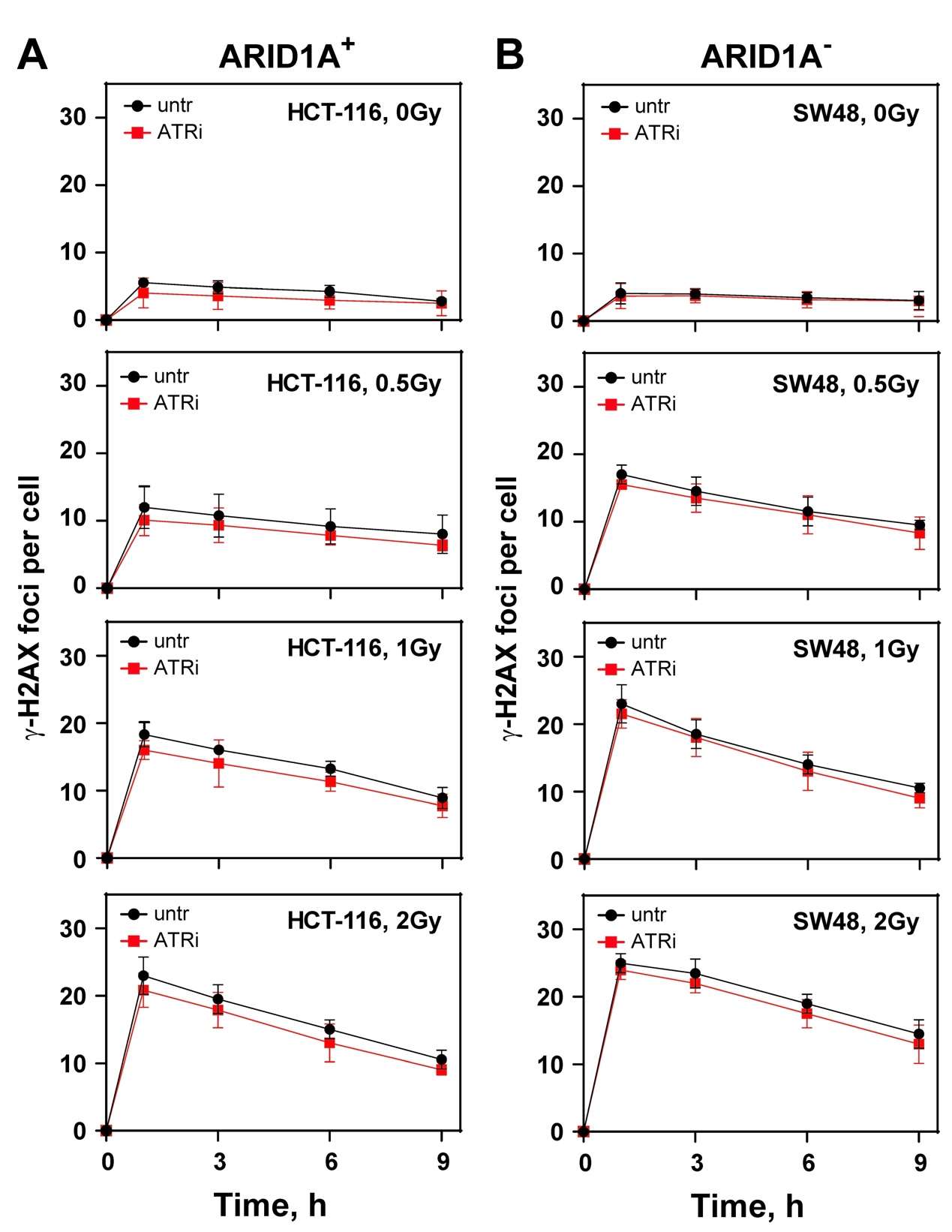


**Figure S10: Kinetics of** γ**H2AX foci formation and decay in G_2_-cells in ARID1A^+^ and ARID1A^-^ CRC cells exposed to 0 Gy, 0.5 Gy, 1.0 Gy and 2.0 Gy.**

A: Kinetics of γH2AX foci formation and decay in ARID1A^+^ cells. B: Kinetics of γH2AX foci formation and decay in ARID1A^-^ cells (n=3).

**
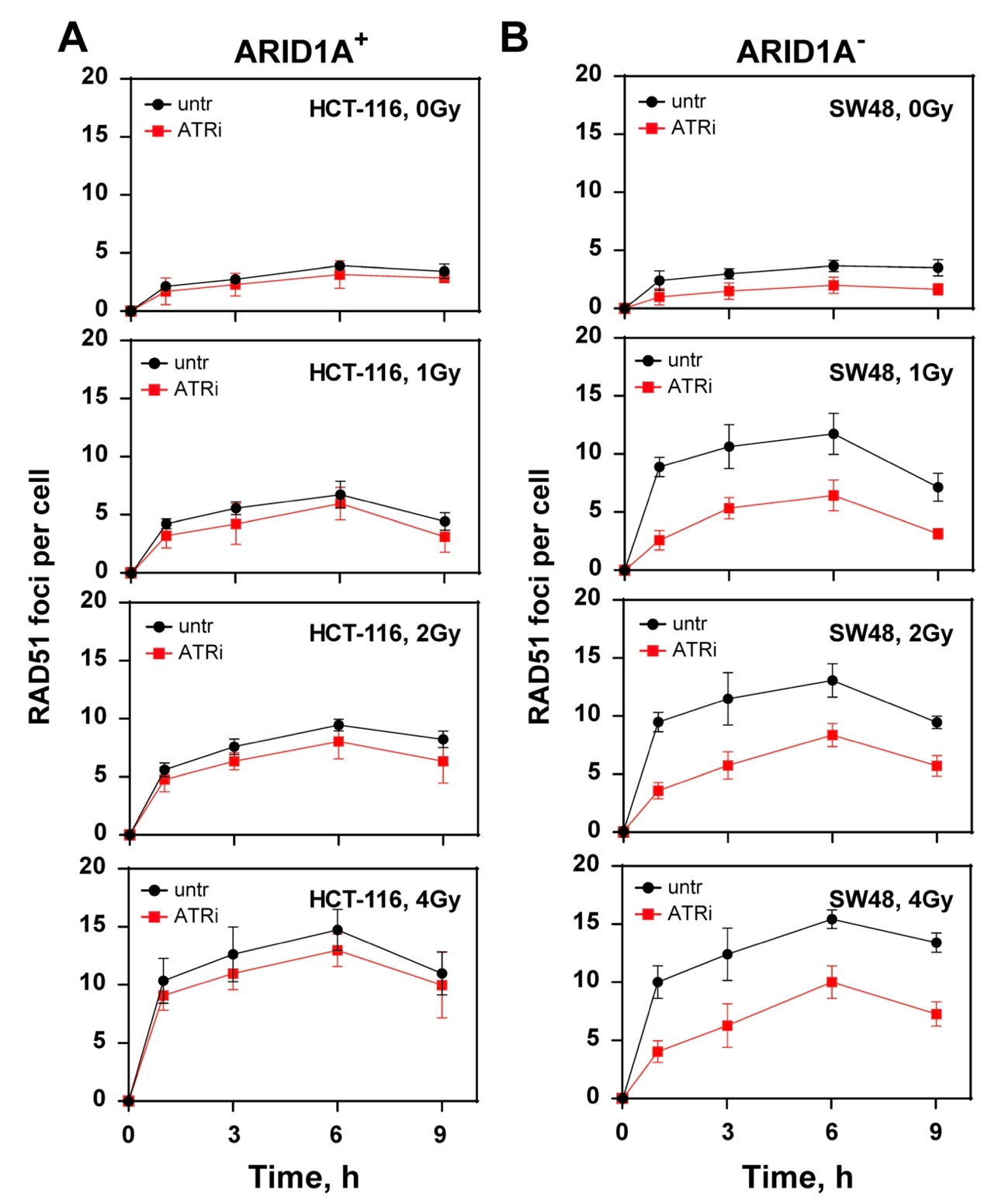
**

**Figure S11: Kinetics of RAD51 foci formation and decay in G2-cells in ARID1A^+^ and ARID1A^-^ CRC cells exposed to 0 Gy, 0.5 Gy, 1.0 Gy and 2.0 Gy.**

A: Kinetics of RAD51 foci formation and decay in ARID1A^+^cells. B: Kinetics of RAD51 foci formation and decay in ARID1A^-^ cells (n=3).

**

**

**Figure S12: Effect of ATRi on RAD51 foci formation in G2-phase CRC cell lines.**

The respective numbers of RAD51 foci at tmax (6h) in G2-phase ARID1A^+^ and ARID1A^-^ cells without (untr) and with 20 nM VE822 (ATRi) after exposure to the indicated IR doses. Results of 3 independent experiments are shown for CRC cell lines (N=3).

**Figure S13: ATRi effect on HR
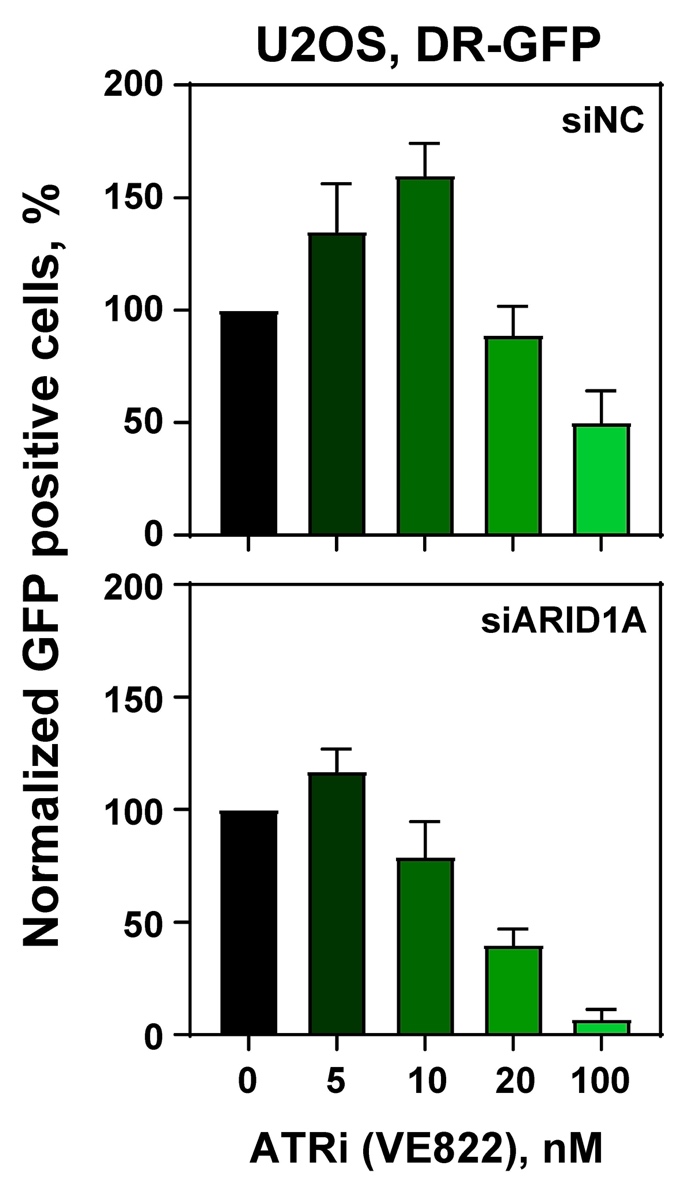
R in ARID1A^+^ U2OS/DR-GFP reporter cells and ARID1A^KD^ U2OS/DR-GFP reporter cells**.

ATRi: VE822, concentration from 5nM to 100nM. U2O2/DR-GFP cells were firstly transfected with siRNA for knock-down of ARID1A expression, and then U2O2/DR-GFP positive cells are measured 24h after I-SceI transfection in untreated cells, as well as in cells treated with VE822 from 0 to 100 nM. Results shown are normalized to those of untreated controls (n=3).
